# Impact of gamma irradiation and guava leaf extract on the quality and storage stability of chicken patties

**DOI:** 10.1002/fsn3.3174

**Published:** 2023-06-22

**Authors:** Anam Sadiq, Muhammad Sajid Arshad, Roshaan Bint Amjad, Haroon Munir, Madiha Rohi, Waseem Khalid, Muhammad Tahir Nadeem, Hafiz Ansar Rasul Suleria

**Affiliations:** ^1^ Department of Food Science, Faculty of Life Sciences Government College University Faisalabad Pakistan; ^2^ Fatima Jannah Medical University Lahore Pakistan; ^3^ Department of Food Science and Technology Government College Women University Faisalabad Faisalabad Pakistan; ^4^ University Institute of Food Science and Technology The University of Lahore Lahore Pakistan; ^5^ Grand Asian University Sialkot Sialkot Pakistan; ^6^ School of Agriculture and Food, Faculty of Veterinary and Agricultural Sciences The University of Melbourne Parkville Vic. Australia

**Keywords:** chicken patties, gamma irradiation, guava leaves extract, microbial, physicochemical parameters, sensory analysis

## Abstract

The current investigation was carried out to evaluate the impact of gamma irradiation and guava leaf extract (GLE) on chicken meat patties. The effects of treatments on chicken meat patties were determined by physicochemical, stability (oxidative and microbial), and antioxidant status during different packaging (aerobic and vacuum) at storage intervals (0, 5, and 10 days). The changes in physicochemical parameters of chicken patties were observed on various treatments, storage intervals, and different packaging. The TBARS and POV were found to increase significantly (*p* < .05) on 2 kGy and with the passage of storage time. The results of microbial load in samples were found to decrease on gamma irradiation with and without GLE. The antioxidant profile in chicken patties was with respect to control. Slight changes were seen in sensory parameters on different treatments at storage intervals. It is concluded that gamma irradiation eliminated the microbes and different concentrations of GLE improve the stability and antioxidant profile of chicken patties.

## Graphical abstract

The current investigation was carried out to evaluate the impact of gamma irradiation and guava leaf extract (GLE) on chicken meat. The effects of treatments on chicken patties were determined by physicochemical, stability, and antioxidant status during different packaging at storage intervals. The changes in physicochemical parameters of chicken patties were observed on various treatments, storage intervals, and different packaging. The TBARS and POV were found to increase on 2 kGy and with the passage of storage time. The results of microbial load in samples were found to decrease on gamma irradiation with and without GLE. The antioxidant profile in chicken patties was with respect to control. Slight changes were seen in sensory parameters on different treatments at storage intervals. It is concluded that gamma irradiation eliminated the microbes and different concentrations of GLE improve the stability and antioxidant profile of chicken patties.

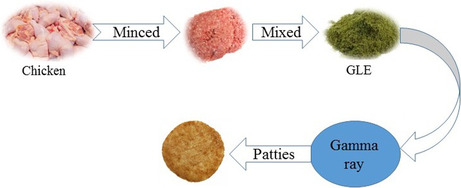

## 1 INTRODUCTION

The safety and quality of meat are current challenges for the production of valuable products. There are various nonthermal processing techniques being used to produce safe meat (Doulgeraki et al., 2012). Current findings in food microbiology have focused on the occurrence and prevalence of emerging foodborne pathogens like Uropathogenic *E. coli*, *Enterococcus faecalis*, *Klebsiella pneumonia*, and *Staphylococcus* in meat and poultry that do not induce respiratory infections (Davis et al., 2015). It is estimated that the number of pathogens capable of causing foodborne infection is 31. These foodborne pathogens are found sometimes present in supermarket raw meats. The nature and quantity of the existing pathogens, however, vary according to the type of meat (Scallan et al., 2011). During handling, bleeding, and processing, meat and poultry products can be affected by external sources. The pollutants on the cutters will spread to different parts of the meat eventually (Arshad, Nisar, et al., 2019).

The chicken meat is needed to be kept safe from microbial growth during capturing, processing, transport as well as storage (Rouger et al., 2017) Chicken meat retains a variety of bacterial groups known as microbiota after these procedures occur. Proliferation and associations between microbiota members contribute to poultry meat degradation (Remenant et al., 2015). Several attempts have been made for decades to classify bacterial consequences arising from meat contamination; as a result, this has been suggested that meat microbiota is the result of microbial interactions between two major bacterial groups: specific spoilage organisms (SSOs) and spoilage‐associated organisms (SAOs) (Boziaris & Parlapani, 2017).

The initial contemporary method for the protection of sustenance was thermal food processing. These maintenance strategies allowed necessary changes, which included protein coagulation, smell development, surface relaxing, and expanding of starch (Proctor et al., 2018). Radiation processing is one of the recently evolving techniques to ensure the viral security of meat (Kanatt et al., 2005). Several techniques have been used by the food industry for decades to store food or to extend the shelf life of food. Irradiation is one of the best modern strategies to achieve microbiological safety without decreasing the nutrient content and sensory predictability of meat and poultry products. The benefits of irradiation are removing pathogens and enhancing the shelf life of meat products (Nam et al., 2004).

To improve the consistency and firmness of chicken and its derivatives, the mixture of gamma irradiation with certain bioactive compounds is advantageous. The dose of gamma irradiation may also be good for reducing it (Arshad, Amjad, et al., 2019). Plants and herb extracts have served a great deal in this regard. Food substances are normally consumable and it needs security against microorganisms through distribution, preparation, and storage.

Guava (*Psidium guajava L*.) is among the most popular fruit trees commonly cultivated in the world's tropical and temperate areas and is considered to be a nutritionally valuable and remunerative crop (Parvez et al., 2018). Guava leaf contains phytochemical antioxidants, for example, lycopene present in the palatable segment of the natural product which is shaded pink. It also contains vitamin C, copper, vitamin B3 (niacin), iron, vitamin A, B6, saponin, tannin, alkaloid, flavonoid, flavonol, cardiac glycosides, morin‐3‐O‐arabinoside, quercetin‐3‐O‐arabinoside phenol, morin‐3‐O‐lyxoside, and dietary fiber (Olaniyan, 2017). Their high degree of antibacterial activity lays a prospective role against *Staphylococcus aureus*, *Escherichia coli*, and *Candida albicans* (Abdel Rahim et al., 2002). In general, its polyphenolic compounds, such as ferulic, protocatechuic, gallic, caffeic acid, and quercetin are correlated with the biological activities of guava (Rai et al., 2010).

The present study was designed to evaluate the effects of different concentrations of guava leaf extract and gamma irradiation on the physicochemical, stability (oxidative and microbial), and antioxidant profile of chicken patties. Different concentrations of guava leaf extract (1%, 2%, 3%) were applied with and without 2 kGy gamma irradiation under different storage conditions (aerobic and vacuum) and time intervals (0, 5, and 10 days).

## 2 MATERIALS AND METHODS

### 2.1 Procurement of materials

All raw materials including chicken and guava leaves were acquired from a local market in Faisalabad, Pakistan. This experimental study was conducted at NIAB, Faisalabad, Pakistan and Department of Food Science, Government College University, Faisalabad, Pakistan. All the analytical grade chemicals and glassware for more research were used and procured from Sigma‐Aldrich®, USA.

#### 2.1.1 Extraction of guava leaf extract

Powdered samples of guava leaves were taken at a solid to solvent ratio of 1:10 in a 500 ml beaker with 70% ethanol. Using an ultrasonic probe (VCX‐750, Sonics and Materials), the separation was carried out. The frequency was calculated at 20 kHz with an amplitude of 50%. The extraction was conducted for 10 min at ambient temperature (Liu et al., 2014). By using Whatman filter paper, the mixture derived from extractions was filtered separately. A rotary evaporator connected to a vacuum pump as well as a refrigerated cooling system were used to evaporate the filtrate from all samples. The total solvent at a vacuum condition of 0.07 MPa was dried at a water bath temperature of 79°C.

### 2.2 Development of chicken patties and gamma irradiation

Minced chicken was molded into patties weighing 50 g each. There were total seven treatments, control, 1% GLE, 2% GLE, 3% GLE, 2kGy + 1% GLE, 2kGy + 2% GLE and 3% GLE + 2kGy. The patties were stored at refrigeration temperature for a period of 10 days in tightly sealed plastic bags (100 gauge). The different testings were performed at intervals of 5 days. The samples were gamma irradiated by using the facility at Nuclear Institute for Agriculture and Biology, Faisalabad‐Pakistan.

### 2.3 Antioxidant profile

#### 2.3.1 Determination of Total phenolic contents (TPC)

The total phenolic content of treated chicken patties was measured by using Folin–Ciocalteu reagent and using a spectrophotometric method (Tezcan et al., 2009). For 0.5 ml of the known sample concentration, 1 ml of the 10% Folin–Ciocalteu assay was briefly added. Before adding 2 ml of 20% sodium carbonate solution to the above solution, the mixture was mixed well and stored for 6 min. Using a spectrophotometer, the phenols were measured at 760 nm after responding at 30°C for 60 min. Using an ordinary Gallic acid solution, a standard curve was prepared and the findings of total phenols were interpreted as 1 g of Gallic acid equivalent (GAE) per gram of substance.

#### 2.3.2 DPPH (2,2‐diphenyl‐1‐picrylhydrazyl) scavenging assay

DPPH (2,2‐diphenyl‐1‐picrylhydrazyl) is a stable, highly colored free radical capable of removing labile hydrogen atoms to polyphenolic compounds with colorless hydrazine (DPPH‐H) associated form (Diouf et al., 2009). Treated patties of the free radical scavenging activity (FRSA) of its GLE were analyzed with certain modifications. In the freshly made DPPH reagent, 0.1 ml of homogenized solution was added and kept for 60 min at room temperature in the dark. The absorbency was determined spectrophotometrically at 517 nm. The maximum FRSA of each specimen was exhibited as a reduced percentage of DPPH and computed as follows: FRSA = 100‐(initial absorbance‐final absorbance)/initial absorbance DPPH absorbance values at time zero and after 60 min.

$\text{FRSA}=\left(\text{initial absorbance}-\text{final absorbance}\right)/\text{initial absorbance}\times 100.$

#### 2.3.3 Ferric reducing antioxidant power (FRAP) assay

The FRAP value of treated chicken was determined by the described method of Benzie and Strain (1996). 30 μl of distilled water and 300 μl of FRAP assay were applied to 10 μl of the homogenized sample; the resulting solution was cultured for 5 min at room temperature. The spectrophotometric use of the Jenway UV–VIS Spectrophotometer at 593 nm was calculated at that stage just after the power of the blue‐hued complex emerged. Mixing 25 ml of Acetate buffer, 2.5 ml of TPTZ (10 mM 2,4,6‐Tripyridyl‐s‐triazine at 40 mM HCl), and (300 mM Sodium acetate at pH 3.6) with 2.5 ml of Ferric chloride solution (20 mM FeCl3.6H2O in distilled water) was used to prepare the FRAP Reagent. Before use, FRAP precipitate was prepared fresh and warm at 37°C. The reducing strength of each sample was calculated at a certain point to be equal to that of 1 mM (FRAP unit) of Fe (II).

### 2.4 Physiochemical analysis of GLE & chicken treated patties

#### 2.4.1 Total volatile basic nitrogen (TVB‐N)

The TVBN assessment was conducted by Karim (2011), with slight modifications by Malle and Tao (1987). Samples were blended at a rate of 1:2 (w/v) with trichloroacetic acid (TCA) and homogenized by using a blender (Waring Industrial Blender) at speed 2. Tests (Centrifuge 5430R Eppendorf AG) were centrifuged for 5 min at 1100 *g* and processed by Whatman No.1 filter paper. In the Kjeldahl processing chamber, 25 ml of the test was pipetted and 5 ml of 10% sodium hydroxide was applied to the solution. Using BUCHI Distillation Unit K‐350, Switzerland, steam processing was conducted.

#### 2.4.2 Hunter color (lab)

With the aid of measurements that were consistent with the calibrated plate (L = 89.2, a = 0.921, and b = 0.783), the surface color values of the GLE‐treated chicken meat patties were measured by the Hunter colorimeter. To use an average of 9 random observations, the CIE L (lightness), CIE a(redness), and CIE b (yellowness) color values were collected on the surface of every sample for statistical analysis.

#### 2.4.3 Heme pigment

The pigments of chicken meat were observed according to the modified method of Warriss, (1979). Four grams of meat product was mixed with 20 ml cold phosphate buffer (40 mM), maintained at pH 6.8, and homogenized at 20,800 *g* for 10 s. The ultraviolet–visible (UV/VIS) spectrometer (Irmeco, u2020 Germany) was used to measure the absorbance at different wavelengths 525, 545, 565, and 572 nm.

The value of myoglobin, oxymyoglobin, and metmyoglobin was calculated according to the below‐given formula:

$\left[\mathrm{Mb}\right]=\left(0.369R1+1.140R2–0.941\text{R3}+0.015\right)\times 100.$


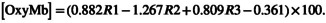



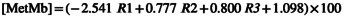
Where, absorbance ratios of *R*1 = *A*572*/A*525, *R*2 = *A*565/*A*525, and *A*545/*A*525.

### 2.5 Stability parameters

#### 2.5.1 Thiobarbituric reactive substances (TBARS)

The outlines characterized by (Schmedes & Hølmer, 1989) described the 2‐thiobarbituric destructive (TBA) values. The 5 g of sample was combined with 25 ml of 20% trichloroacetic acid (200 g/L) in a 30‐s homogenizer solution of 135 ml/L phosphoric acid. Via filter paper (Whatman number 4) the homogenized samples were isolated to discard solid particles from the filtrate. 2 ml of 0.02 M watery TBA solution (3 g/L) was applied to 2 ml of filtrate in a glass container at that stage. Test containers were cultured at 100°C for 30 min from that point onwards and cooled in flowing tap water. The absorption of supernatant substances using the UV–VIS spectrophotometer has been measured at 532 nm. TBA parameters were recorded from a standard curve and stated as milligram malonaldehyde per kilogram (MA/kg) of a sample.

$$\mathrm{mg},\text{malondialdehydes}\;\mathrm{per}\;\mathrm{kg}\;\text{meat}=\frac{\left(\text{Sample absorbance}–\text{blank}\right)\times \text{Total sample}\;\mathrm{vol}.}{0.000156\times 1000}$$

#### 2.5.2 Peroxide value (POV)

The POV value of treated chicken meat patties was measured according to described method of (Sallam et al., 2004) with few modifications. The 3 g samples were measured in the Erlenmeyer flask then warmed inside a water bath to vaporize the fat for 3 min at 60°C. The jar was then agitated with 30 ml of acetic acid–chloroform solution (3:2 v/v) for 3 min to split down the fat. To extract the solid particles from the filtrate, Whatman channel paper number 1 was used. The process was carried out with the addition of starch as an indicator just after the addition of potassium iodide (0.5 ml) for filtration. Titration proceeded against the normal sodium thiosulfate solution. Using the following equation, POV was measured as milliequivalent peroxide per sample kilogram.

$\mathrm{POV}\;\left(\mathrm{meq}/\mathrm{kg}\right)=\left\{\left(S\times N\right)/W\right\}\times 100.$

where N is the normality of sodium thiosulfate solution (N = 0.01), S is the volume of titrant (ml), and W is the weight of the sample (g).

### 2.6 Microbial analysis

#### 2.6.1 Total bacterial count

The 90 ml maximum recovery diluent (MRD) was homogenized for a total of 10 ± 0.1 g of patties mixture. Following adequate dilution, a different concentration was prepared. Using a sterile glass spreader, 0.1 ml of the concentration was spread correctly on plate count agar (PCA). According to (Linton & Boersma, 2003) and (Karim, 2011), maximum bacterial counts have been carried out. The counts of bacteria were expressed per gram of sample as log‐forming units (log_10_CFU g‐1).

### 2.7 Sensory evaluation of chicken patties

The patties prepared from chicken meat were subjected to sensory evaluation. Trained panelists measured the sensory properties by using 9‐point hedonic response scale (9 = like extreme to 1 = extremely dislike). To determine flavor, color, taste, odor, and texture, all panelists were given mineral water and gave their results on the evaluation sheet (Meilgaard et al., 2007).

### 2.8 Statistical analysis

The results obtained from each parameter were analyzed statistically by using Statistic 8.1 (ANOVA). Significance levels (5% alpha) were tested by following the principles established by Steel and Torrie (2012) using 3 factorial factors under CRD. Three replicates were used in all parameters beside of sensory and Hunter color analysis.

## 3 RESULT AND DISCUSSION

### 3.1 Determination of antioxidant potential of chicken sample

#### 3.1.1 Determination of total phenolic contents (TPC)

Phenolic is among the primary compounds that act as essential antioxidants or terminators of free radicals. The measurement of maximum phenolic compounds is one of the critical criteria for estimating the number of antioxidants. Our TPC findings of chicken meat patties were found to alter significantly with respect to treatments and storage intervals. The maximum value of TPC (74.20 ± 0.40 mg/g) was obtained when treated with 3% GLE in aerobic packaged sample at 0 days of storage. However, at vacuum packaged sample value was 78.65 ± 0.42 mg/g when treated with 3% GLE at 0 days of storage whereas, the lowest value was found in aerobic and vacuum packaged samples on 0 days of storage when treated with 2 kGy + 3% GLE (Table 1). Moreover, on the 10th day, the higher TPC (65.76 ± 0.42 mg/g) was found in treated 3% GLE aerobic packaged samples, and extreme value of vacuum packaged sample was 69.43 ± 0.46 mg/g when treated 3% GLE at 10 days of storage, however, the lower value was observed for vacuum packaged samples at 10 days of storage when treated with 2 kGy + 3% GLE and in aerobic packaged samples at 10 days of storage when treated with the control group in chicken meat patties.

**TABLE 1:** Total phenolic content (TPC) of chicken patties treated with gamma irradiation and guava leaf extract at different storage periods (0, 5, and 10 days).

Parameter	Irradiation dose (kGy)	Storage period (day)					
		0		5		10	
		Aerobic	Vacuum	Aerobic	Vacuum	Aerobic	Vacuum
TPC (mg/g)	Control	55.23 ± 0.42	60.21 ± 0.45	51.75 ± 0.30	56.24 ± 0.40	46.29 ± 0.32	51.11 ± 0.32
	GLE1%	64.15 ± 0.34	68.75 ± 0.42	61.23 ± 0.25	63.14 ± 0.43	59.24 ± 0.25	60.05 ± 0.56
	GLE2%	69.76 ± 0.34	72.24 ± 0.50	65.12 ± 0.32	68.54 ± 0.30	60.20 ± 0.32	62.45 ± 0.50
	GLE3%	74.20 ± 0.40	78.65 ± 0.42	69.12 ± 0.41	73.41 ± 0.25	65.76 ± 0.42	69.43 ± 0.46
	2 kGy + 1% GLE	62.75 ± 0.46	65.75 ± 0.39	60.25 ± 0.58	62.39 ± 0.27	56.23 ± 0.28	58.61 ± 0.32
	2 kGy + 2% GLE	59.05 ± 0.42	63.05 ± 0.30	57.75 ± 0.34	61.75 ± 0.31	52.01 ± 0.30	57.25 ± 0.50
	2 kGy + 3% GLE	54.82 ± 0.31	58.24 ± 0.25	51.68 ± 0.36	53.25 ± 0.31	46.25 ± 0.30	50.21 ± 0.32
	Mean	62.85 ± 0.38b	66.69 ± 0.39a	59.55 ± 0.36b	62.67 ± 0.32a	55.14 ± 0.31b	58.44 ± 0.42a

*Note*: The values are mean ± SD of three independent determinations. Means carrying different letters in a column differed significantly (*p* < .05).

Abbreviation: GLE, guava leaf extract.

The findings showed that, relative to aerobic samples, the TPC was found to increase significantly in vacuum‐packaged chicken meat patties. Another research showed that *S. aromaticum* was found to have the highest amount of TPC. Although *C. cassia* was found to have the lowest amount in raw chicken meat (Qwele et al., 2013). The depicted results of Arshad, Amjad, et al. (2019) revealed that turmeric powder with chicken patties display higher antioxidant capacity. Habeeb et al. (2007) reported that throughout the storage period, the TPC of Aloe vera‐treated nuggets raised slightly (*p* < .05); furthermore, the values are generally (*p* < .05) lower than that of control nuggets on all storage days. The antimicrobial effect of aloe vera may be attributed to a relatively slow increase in the TPC of aloe vera‐treated nuggets. Another research showed a similar effect that the bioactive ingredients liable for the antimicrobial activity of certain antioxidant products can be due to a relatively slow change in the TPC of treated items (Candogan, 2002; Lansky & Newman, 2007; Nawaz et al., 2006). Similarly, another study stated by Ekaluo et al. (2015) demonstrated significantly greater total phenolic content than the bitter leaf (*Vernonia amygdalina*) in guava leaf care. Kim et al. (2013) reported similar results in meat patties, which also observed a corresponding decrease in the TPC of tomato powder‐treated products.

#### 3.1.2 DPPH (2,2‐diphenyl‐1‐picrylhydrazyl) scavenging assay

The free radical scavenging method of 2,2‐Diphenyl‐1‐picrylhydrazyl (DPPH) is a technique for evaluating the antioxidant potential of food and food products. This is now the modest technique where the eventual component and perhaps extract is mixed with the solution of DPPH and absorption is reported after a defined period (Yaqoob et al., 2020).

DPPH (2,2‐diphenyl‐1‐picrylhydrazyl) value of guava leaf extract and gamma irradiation‐treated chicken meat patties were found to change significantly at both packaging and storage intervals. A higher value of DPPH (64.23% ± 0.40%) was observed on GLE 3% aerobically packaged samples at 0 days of storage. However, in vacuum packaged patties, the value was 69.11% ± 0.39% when treated with 3%GLE at 0 days of storage, while the lowest value was measured in aerobic and vacuum packaged samples at 0 days of storage when treated with a control group and 2 kGy + GLE3%. Moreover, at 10 days of storage, higher DPPH (59.04% ± 0.42%) was found in 3%GLE‐treated chicken meat patties (aerobic packaged), followed by vacuum packaged sample value (61.23% ± 0.50%) when treated with 2%GLE while the lower value was observed for vacuum packaged samples on day 10 of storage when treated with 2 kGy + 3% GLE and in aerobic packaged samples, the lower value was found in the control group of chicken meat patties (Table 2).

**TABLE 2:** 2,2‐diphenyl‐1‐picrylhydrazyl (DPPH) of chicken patties treated with gamma irradiation and guava leaf extract at different storage periods (0, 5, and 10 days).

Parameter	Irradiation dose (kGy)	Storage period (day)					
		0		5		10	
		Aerobic	Vacuum	Aerobic	Vacuum	Aerobic	Vacuum
DPPH (%)	Control	51.42 ± 0.42	61.05 ± 0.45	48.23 ± 0.30	58.21 ± 0.40	45.21 ± 0.32	55.01 ± 0.32
	1% GLE	55.56 ± 0.34	65.23 ± 0.42	53.73 ± 0.25	62.01 ± 0.43	50.01 ± 0.25	60.46 ± 0.56
	2% GLE	58.97 ± 0.34	67.21 ± 0.50	55.21 ± 0.32	65.45 ± 0.30	51.04 ± 0.32	61.23 ± 0.50
	3% GLE	64.23 ± 0.40	69.11 ± 0.39	62.01 ± 0.41	63.23 ± 0.25	59.04 ± 0.42	59.04 ± 0.46
	2 kGy + 1% GLE	60.45 ± 0.36	63.42 ± 0.39	58.23 ± 0.58	59.01 ± 0.27	55.01 ± 0.28	57.03 ± 0.32
	2 kGy + 2% GLE	59.01 ± 0.32	61.04 ± 0.30	56.23 ± 0.34	57.25 ± 0.31	54.23 ± 0.30	55.11 ± 0.50
	2 kGy + 3% GLE	57.21 ± 0.28	58.29 ± 0.25	55.04 ± 0.36	56.11 ± 0.31	51.11 ± 0.30	52.04 ± 0.21
	Mean	58.12 ± 0.35b	63.62 ± 0.38a	55.53 ± 0.36b	60.18 ± 0.32a	52.23 ± 0.31b	57.13 ± 0.41a

*Note*: The values are mean ± SD of three independent determinations. Means carrying different letters in a column differed significantly (*p* < .05).

Abbreviation: GLE, guava leaf extract.

Our results showed that the DPPH value increased significantly with an increased concentration of GLE while the DPPH value reduced with the passage of time. In addition, the DPPH value in un‐treated chicken samples (control) also declined with the flow of time. Results showed that during 6 days of cold storage, the Bidenspilosa leaf extract exhibited greater antiradical interaction toward 2,2‐diphenyl‐2‐picrylhydrazyl (DPPH) or 2,2íazinobis‐3‐ethylbenzothiazoline‐6‐sulfonic acid (ABTS) radicals compared to Moringaoleifera leaves extract as well as normal butylated hydroxy toluene (BHT) renewed ground beef (Falowo et al., 2017).

The findings of our research are in accordance with the suggestion of Fernandes et al. (2018) who observed the antioxidant capacity of spray‐dried guava leaves extracts. Imran et al. (2017) recorded the same effect of antioxidants through wheat germ oil or alpha‐lipoic acid in developed chicken and the maximum inhibition of DPPH was found in the developed nuggets. Our results are similar to Yaqoob et al., 2020 who proved that with the passage of time, the DPPH values decreased significantly across all treatments.

#### 3.1.3 Ferric reducing antioxidant power (FRAP) assay

The ferric reducing/antioxidant power is one of the most frequently used techniques for evaluating the appropriate antioxidant power of a substance. The guava leaf extract and gamma irradiation‐treated chicken meat patties observed significantly regarding storage intervals, packaging, and treatments.

The higher value of FRAP (5.34 ± 0.03 μM TE g‐1) was found in treated 3% GLE in aerobic packaged samples at 0 days of storage. The higher value of vacuum packaged sample was 5.61 ± 0.03 μM TE g‐1 when treated with 3% GLE at 0 days of storage, whereas the lower value of aerobic and vacuum packaged samples was 3.12 ± 0.02 μM TE g‐1 and 4.23 ± 0.01 μM TE g‐1, respectively (Table 3). Even though at 10 days the higher FRAP (4.91 ± 0.03 μM TE g‐1) was observed in treated 3% GLE in aerobic packaged samples and in vacuum packaged samples the value was 5.14 ± 0.03 μM TE g‐1 when treated with 3% GLE. However, the lower FRAP value of aerobic and vacuum packaged samples was 2.55 ± 0.01 μM TE g‐1 and 3.52 ± 0.02 μM TE g‐1, respectively.

**TABLE 3:** Ferric reducing antioxidant power (FRAP) of chicken patties treated with gamma irradiation and guava leaf extract at different storage periods (0, 5, and 10 days).

Parameter	Irradiation dose (kGy)	Storage period (day)					
		0		5		10	
		Aerobic	Vacuum	Aerobic	Vacuum	Aerobic	Vacuum
FRAP (μM TE g‐1)	Control	3.12 ± 0.02	4.23 ± 0.01	2.82 ± 0.01	3.90 ± 0.02	2.55 ± 0.01	3.52 ± 0.02
	1% GLE	4.70 ± 0.02	4.97 ± 0.02	4.35 ± 0.02	4.57 ± 0.02	4.12 ± 0.02	4.35 ± 0.03
	2% GLE	4.91 ± 0.03	5.25 ± 0.04	4.75 ± 0.02	5.04 ± 0.04	4.35 ± 0.02	4.64 ± 0.02
	3% GLE	5.34 ± 0.03	5.61 ± 0.03	5.02 ± 0.03	5.31 ± 0.05	4.91 ± 0.03	5.14 ± 0.03
	2 kGy + 1% GLE	5.15 ± 0.04	4.83 ± 0.03	4.69 ± 0.03	4.56 ± 0.03	4.24 ± 0.03	4.02 ± 0.02
	2 kGy + 2% GLE	4.80 ± 0.03	4.51 ± 0.03	4.51 ± 0.02	4.42 ± 0.02	4.30 ± 0.03	4.16 ± 0.02
	2 kGy + 3% GLE	4.56 ± 0.02	4.29 ± 0.02	4.32 ± 0.02	3.89 ± 0.02	3.91 ± 0.02	3.59 ± 0.01
	Mean	4.65 ± 0.03b	4.81 ± 0.02a	4.35 ± 0.02b	4.53 ± 0.03a	4.05 ± 0.02b	4.20 ± 0.02a

*Note*: The values are mean ± SD of three independent determinations. Means carrying different letters in a column differed significantly (*p* < .05).

Abbreviation: GLE, guava leaf extract.

The strong preference was noticed to substantially reduce the FRAP values as the storage period passed. The previous outcome showed that guava leaves contained high FRAP values (3.69 ± 0.03 mM FeSO4/mg leaf d.w.). Another research is similar to our finding in which Yaqoob et al. (2020) reported that FRAP values increased significantly with guava leaf extract and papaya leaf extract in shrimp patties.

### 3.2 Physicochemical parameters of chicken

#### 3.2.1 TVBN

A significant measure for determining meat's freshness is the total volatile basic nitrogen (TVB‐N) content. Complete volatile basic nitrogen (TVB‐N), an essential index reflecting the dimethylamine, trimethylamine, and ammonia content in the measured samples, has been used extensively in the quality determination of meat and meat products. The evaluation is a simple way to determine the value of meat products as ammonia creation increases during decomposition due to protein deamination (Howgate, 2010).

The TVBN value of chicken meat patties was observed to change with respect to different storage, treatments, and packaging. The higher TVBN was 17.15 ± 0.45 mg Nitrogen/100 g that was observed on 2 kGy + 3% GLE (aerobic packaged samples) at 0 days of storage. Moreover, the high value of vacuum packaged samples was 13.26 ± 0.39 mg Nitrogen/100 g when treated with 2 kGy + 3% GLE at 0 days of storage, while the lower of aerobic and vacuum packaged samples were observed at 0 days when treated with 3% GLE. Although, at 10 days, the higher TVBN of aerobic packaged and vacuum packaged samples was 22.18 ± 0.34 mg Nitrogen/100 g and 21.05 ± 0.20 mg Nitrogen/100 g, respectively, at 2 kGy + 3% GLE while the lowest TVBN of aerobic packaged and vacuum packaged samples were 15.05 ± 0.30 mg Nitrogen/100 g and 11.23 ± 0.20 mg Nitrogen/100 g, respectively, when treated with 3% GLE (Table 4). The findings showed that, relative to aerobic samples, the TVBN value decreased expressively in vacuum‐packaged chicken meat patties. The TVBN value also increased when the storage time increased, and a noticeable rise was found in control.

**TABLE 4:** Total volatile basic nitrogen (TVBN) of chicken patties treated with gamma irradiation and guava leaf extract at different storage periods (0, 5, and 10 days).

Parameter	Irradiation dose (kGy)	Storage period (day)					
		0		5		10	
		Aerobic	Vacuum	Aerobic	Vacuum	Aerobic	Vacuum
TVBN (mg Nitrogen/100 g)	Control	11.12 ± 0.30	9.23 ± 0.21	16.01 ± 0.32	12.05 ± 0.48	21.05 ± 0.32	16.04 ± 0.32
	1%GLE	8.50 ± 0.20	7.15 ± 0.24	13.01 ± 0.30	11.08 ± 0.40	17.51 ± 0.20	14.15 ± 0.21
	2%GLE	7.05 ± 0.10	6.01 ± 0.42	9.18 ± 0.34	9.05 ± 0.31	15.25 ± 0.32	12.25 ± 0.20
	3%GLE	5.23 ± 0.50	5.13 ± 0.31	11.04 ± 0.32	9.01 ± 0.20	15.05 ± 0.30	11.23 ± 0.20
	2 kGy + 1%GLE	10.01 ± 0.23	8.26 ± 0.35	13.25 ± 0.30	12.04 ± 0.35	18.05 ± 0.28	15.01 ± 0.23
	2 kGy + 2%GLE	13.21 ± 0.3	10.01 ± 0.50	15.65 ± 0.21	13.21 ± 0.31	19.26 ± 0.24	15.05 ± 0.25
	2 kGy + 3%GLE	17.15 ± 0.45	13.26 ± 0.39	20.24 ± 0.36	16.01 ± 0.45	22.18 ± 0.34	21.05 ± 0.20
	Mean	10.32 ± 0.29a	8.44 ± 0.34b	14.05 ± 0.31a	11.78 ± 0.36b	18.33 ± 0.28a	14.97 ± 0.23b

*Note*: The values are mean ± SD of three independent determinations. Means carrying different letters in a column differed significantly (p < .05).

Abbreviations: GLE, guava leaf extract.

Latou et al. (2014) stated that the TVBN meat indicator comes primarily from the processing and decomposing of proteins as well as other nitrogen‐containing compounds with a microbial metabolism feature. Ayari et al. (2016) demonstrated that with the storage interval, the TVBN content increased significantly, but radiation treatment suppressed the accumulation of TVBN during storage time. Our results showed the similarity with Dogruyol and Mol (2017) who reported that, without radiation treatment, TVBN increased at 0 days, but because of irradiated samples, the TVBN value decreased as compared to nonirradiated samples during the storage period of 40 days.

Yaqoob et al. (2020) conducted a study on shrimp patties treated with GLE and PLE. TVBN value was found to increase with the passage of time while this value was observed to reduce with GLE and PLE concentration. Another research by Arshad, Amjad, et al. (2019) depicted that in vacuum‐packaged chicken meat, the TVBN value decreased significantly due to metabolic samples. In addition, the TVBN value enlarged with the passage of time in unprocessed chicken meat (control).

### 3.3 Stability analysis of chicken

#### 3.3.1 Thiobarbituric reactive substances (TBARS)

Thiobarbituric reactive substances is the oldest and one of the most widely used methods to calculate malondialdehyde. Significant variations were observed in the TBARS value of chicken meat patties on different treatments, packaging, and storage.

The TBARS value in chicken meat patties ranged from 0.20 ± 0.02 MDA/kg to 0.40 ± 0.04 MDA/kg. The higher TBARS value was found on 2 kGy + 1% GLE while the lower value was observed on 3% GLE as shown in Table 5. During storage, with the passage of time the TBARS value was recorded to high. However, at 10 days, high TBARS value was found while a lower value was observed at 0 days of storage. Our result show that packaging materials affect the TBARS value of chicken meat patties. Moreover, maximum TBARS value was found in aerobic packaged samples, whereas a minimum value was recorded in vacuum packaged samples.

**TABLE 5:** Thiobarbituric acid reactive substances (TBARS) value of chicken patties treated with gamma irradiation and guava leaf extract at different storage periods (0, 5, and 10 days).

Parameter	Irradiation dose (kGy)	Storage period (day)					
		0		5		10	
		Aerobic	Vacuum	Aerobic	Vacuum	Aerobic	Vacuum
TBARS (MDA/kg)	Control	0.31 ± 0.02	0.29 ± 0.03	0.32 ± 0.02	0.31 ± 0.02	0.34 ± 0.02	0.36 ± 0.01
	1%GLE	0.28 ± 0.02	0.26 ± 0.02	0.30 ± 0.01	0.29 ± 0.03	0.33 ± 0.02	0.31 ± 0.02
	2%GLE	0.25 ± 0.01	0.23 ± 0.02	0.29 ± 0.03	0.27 ± 0.01	0.31 ± 0.02	0.30 ± 0.02
	3%GLE	0.23 ± 0.02	0.20 ± 0.02	0.26 ± 0.01	0.24 ± 0.02	0.30 ± 0.02	0.27 ± 0.02
	2 kGy + 1%GLE	0.32 ± 0.03	0.30 ± 0.02	0.34 ± 0.02	0.33 ± 0.03	0.40 ± 0.04	0.39 ± 0.04
	2 kGy + 2%GLE	0.30 ± 0.02	0.28 ± 0.02	0.32 ± 0.03	0.32 ± 0.01	0.36 ± 0.02	0.34 ± 0.03
	2 kGy + 3%GLE	0.27 ± 0.02	0.24 ± 0.02	0.33 ± 0.02	0.28 ± 0.01	0.38 ± 0.02	0.33 ± 0.02
	Mean	0.28 ± 0.02a	0.26 ± 0.02b	0.31 ± 0.02a	0.29 ± 0.02b	0.34 ± 0.02a	0.33 ± 0.02a

*Note*: The values are mean ± SD of three independent determinations. Means carrying different letters in a column differed significantly (*p* < .05).

Abbreviation: GLE, guava leaf extract.

Our findings showed that the TBARS value was observed to decrease by increasing the GLE percentage, while these values were recorded high when the gamma irradiation dose was applied. However, the TBARS value was found to be high with the passage of storage time. Furthermore, the packaging materials affected the chicken meat patties. Results showed that the aerobic packed samples were contained a high TBARS value as compared to vacuum packed samples (Table 6).

**TABLE 6:** Peroxide value (POV) of chicken patties treated with gamma irradiation and guava leaf extract at different storage periods (0, 5, and 10 days).

Parameter	Irradiation dose (kGy)	Storage period (day)					
		0		5		10	
		Aerobic	Vacuum	Aerobic	Vacuum	Aerobic	Vacuum
POV (meq peroxide/kg)	Control	0.21 ± 0.01	0.19 ± 0.03	0.23 ± 0.03	0.21 ± 0.03	0.27 ± 0.02	0.25 ± 0.03
	1% GLE	0.18 ± 0.02	0.15 ± 0.02	0.22 ± 0.03	0.19 ± 0.02	0.28 ± 0.02	0.23 ± 0.02
	2% GLE	0.15 ± 0.02	0.13 ± 0.02	0.18 ± 0.04	0.16 ± 0.01	0.24 ± 0.03	0.21 ± 0.05
	3% GLE	0.13 ± 0.01	0.12 ± 0.01	0.17 ± 0.03	0.15 ± 0.01	0.26 ± 0.01	0.19 ± 0.02
	2 kGy + 1% GLE	0.23 ± 0.03	0.20 ± 0.03	0.25 ± 0.02	0.23 ± 0.03	0.29 ± 0.03	0.26 ± 0.03
	2 kGy + 2% GLE	0.21 ± 0.02	0.19 ± 0.02	0.23 ± 0.03	0.22 ± 0.03	0.26 ± 0.03	0.24 ± 0.03
	2 kGy + 3% GLE	0.17 ± 0.02	0.13 ± 0.01	0.20 ± 0.03	0.18 ± 0.02	0.25 ± 0.01	0.22 ± 0.02
	Mean	0.18 ± 0.02a	0.16 ± 0.02b	0.21 ± 0.03a	0.19 ± 0.02b	0.26 ± 0.02a	0.22 ± 0.02b

*Note*: The values are mean ± SD of three independent determinations. Means carrying different letters in a column differed significantly (*p* < .05).

Abbreviation: GLE, guava leaf extract.

Our study is similar to Chouliara et al. (2005), who reported that the TBA value in irradiated samples was greater than that in nonirradiated control on the 21st d of storage. Deterioration occurs during the storage of meat‐based substances by rancidity arising from oxidation that takes place for the triacylglycerol molecules at the double bond sites. From previous research, it is clear that the process of oxidation causes significant economic losses for both the food industry and consumers (Kim & Kim, 2017). Our findings showed similarity with Yu et al. (2018), who observed that the TBARS value increased with the storage period. Arshad, Amjad, et al. (2019) reported that the TBARS increases with the treatment of turmeric powder and reduced TBARS value with gamma irradiation dose in chicken meat patties during storage intervals. Furthermore, our results showed similarity with Yaqoob et al. (2020) who treated shrimp patties with GLE and PLE at storage intervals. Our results are correlated with Arshad, Amjad, et al. (2019) who reported that an increase in irradiation amount with the passage of time causes lipid oxidation in chicken meat patties.

#### 3.3.2 Peroxide value (POV)

The Peroxide value is widely used for calculating the state of oxidation in oils and fats as well as its value computes the oxidative rancidity and level of oxidation of the oils/fats (Anjum et al., 2012; Domínguez et al., 2018).

Our POV results show significant changes on different treatments during aerobic and vacuum packaging at different storage. The POV in chicken meat patties ranged from 0.12 ± 0.01 meq peroxide/kg to 0.29 ± 0.03 meq peroxide/kg. The higher POV was found on 2 kGy + 1% GLE while the lower value was observed on 3% GLE. During storage, with the passage of time, the POV was recorded to be high. However, at 10 days, a high POV was found while lower value was observed at 0 days of storage. Our results showed that packaging materials affect the POV of chicken meat patties. Moreover, maximum POV was found in aerobic packaged samples, whereas minimum value was recorded in vacuum packaged samples.

Our results showed that the POV was observed to reduce by increasing the GLE percentage, while POV was recorded high when the gamma irradiation dose was applied. However, the POV was found to be high with the passage of storage time. Furthermore, the packaging materials affected the chicken meat patties. Results showed that the aerobic packed samples contained a high POV as compared to vacuum‐packed samples (Table 6).

The findings showed that, relative to aerobic samples, the POV decreased expressively in vacuum‐packaged chicken meat patties. Although in our research, together at the start or end of the research, the POV in control was significantly high. The impacts of the combined treatment of turmeric powder and irradiation in chicken meat patties were stated by Arshad, Amjad, et al. (2019). The increase in peroxide value was described with the duration of storage time. After irradiation, the increased amount of peroxides occurs because free radicals such as OH‐ and H+ are formed by the radiolysis of water present in meat (Ouattara et al., 2002). In addition, the results of the present study are similar to the findings of Zhao et al. (2017), who stated that due to irradiation and storage interval, increased lipid oxidation was observed.

### 3.4 Microbial analysis

#### 3.4.1 Coliform and total bacterial count

The bacterial counts were evaluated for the maximum aerobic bacteria and coliforms in the untreated and treated (GLE and gamma irradiation) samples. The total aerobic bacteria in chicken meat patties ranged from 3.67 ± 0.25 log CFU/g to 12.5 ± 0.32 log CFU/g. The peak value was found on control treatment, whereas lower TAB was found on 2 kGy + 3% GLE. At different storage intervals, the higher TAB was recorded at 10 days, whereas the lower value was observed at o day. Furthermore, the TAB value of the aerobic packed sample was high with respect to vacuum‐packed samples. Our results showed that the TAB amount was observed to reduce with increasing the GLE percentage and also TAB amount was found to be reduced when treated gamma irradiation dose was applied as shown in Table 7. However, the TAB amount was measured to be high with the passage of storage time. Furthermore, the packaging materials affected the chicken meat patties. Results showed that the aerobic packed samples contained a high TAB amount as compared to vacuum‐packed samples.

**TABLE 7:** Total aerobic bacteria and coliforms of chicken patties treated with gamma irradiation and guava leaf extract at different storage periods (0, 5, and 10 days).

Parameter	Irradiation dose (kGy)	Storage period (day)					
		0		5		10	
		Aerobic	Vacuum	Aerobic	Vacuum	Aerobic	Vacuum
Total aerobic bacteria (log CFU/g)	Control	7.36 ± 0.22	6.48 ± 0.32	9.11 ± 0.30	7.45 ± 0.40	12.56 ± 0.32	9.65 ± 0.32
	1% GLE	6.25 ± 0.14	5.87 ± 0.21	7.98 ± 0.25	6.34 ± 0.13	9.67 ± 0.25	8.34 ± 0.46
	2% GLE	6.01 ± 0.09	5.09 ± 0.16	7.46 ± 0.32	5.87 ± 0.20	8.45 ± 0.32	7.65 ± 0.21
	3% GLE	5.67 ± 0.15	4.37 ± 0.23	6.25 ± 0.41	5.34 ± 0.25	8.05 ± 0.42	6.21 ± 0.16
	2 kGy + 1% GLE	7.18 ± 0.26	6.23 ± 0.39	8.17 ± 0.58	7.54 ± 0.27	10.35 ± 0.28	8.65 ± 0.32
	2 kGy + 2% GLE	5.45 ± 0.18	4.11 ± 0.30	7.28 ± 0.24	6.12 ± 0.31	9.36 ± 0.30	8.21 ± 0.50
	2 kGy + 3% GLE	4.23 ± 0.02	3.67 ± 0.25	6.15 ± 0.36	4.45 ± 0.02	7.54 ± 0.20	5.18 ± 0.32
	Mean	6.02 ± 0.15a	5.12 ± 0.26b	7.58 ± 0.35a	6.15 ± 0.23b	9.43 ± 0.29a	7.69 ± 0.33b
Coliforms (log CFU/g)	Control	4.65 ± 0.07	4.14 ± 0.02	5.25 ± 0.32	4.53 ± 0.24	6.52 ± 0.34	5.12 ± 0.23
	1% GLE	4.12 ± 0.02	3.76 ± 0.06	4.77 ± 0.28	4.32 ± 0.56	5.23 ± 0.32	4.76 ± 0.21
	2% GLE	3.56 ± 0.13	3.31 ± 0.02	3.98 ± 0.26	3.78 ± 0.04	4.23 ± 0.21	4.05 ± 0.15
	3% GLE	3.22 ± 0.16	3.12 ± 0.01	3.76 ± 0.05	3.44 ± 0.02	4.02 ± 0.20	3.78 ± 0.02
	2 kGy + 1% GLE	4.54 ± 0.23	4.12 ± 0.15	4.87 ± 0.21	4.54 ± 0.03	5.32 ± 0.27	5.21 ± 0.35
	2 kGy + 2% GLE	4.41 ± 0.21	4.23 ± 0.18	4.65 ± 0.26	4.45 ± 0.14	4.98 ± 0.26	4.68 ± 0.26
	2 kGy + 3% GLE	3.38 ± 0.08	3.21 ± 0.06	3.74 ± 0.09	3.58 ± 0.02	4.16 ± 0.12	3.99 ± 0.05
	Mean	3.98 ± 0.1a	3.69 ± 0.07b	4.43 ± 0.21a	4.09 ± 0.15b	4.92 ± 0.24a	4.51 ± 0.18b

*Note*: The values are mean ± SD of three independent determinations. Means carrying different letters in a column differed significantly (*p* < .05).

Abbreviation: GLE, guava leaf extract.

Table 7 showed the significant changes of coliform bacteria in chicken meat patties according to different treatments, storage conditions, and packaging. The coliform in chicken meat patties ranged from 3.12 ± 0.01 log CFU/g to 6.52 ± 0.34 log CFU/g. The increment amount was found in aerobic packed sample at 10 days, whereas the lower amount of coliform was observed in vacuum package sample at 0 day. The maximum amount was found in control, whereas lower coliform was found in 3% GLE. Our finding showed that the coliform amount was observed to reduce with enhancing the percentage of GLE and also coliform amount was noted to be reduced when treated with gamma irradiation dose. Moreover, the coliform amount was measured to be high with the passage of storage time. Furthermore, the packaging materials affected the chicken meat patties. Finding showed that the aerobic packed samples were contained a high coliform amount as compared to vacuum‐packed samples.

An et al. (2017) stated that irradiation decreased the bacterial load and increased the storage life of meat products, which is in line with our research findings. Our research is similar to Arshad, Amjad, et al. (2019) who predicted that TAB and coliform amount more in aerobic package sample as compared to vacuum packaging. Another study showed that 6 log_10_ CFU/g was really the acceptable limit of aerobic plate count, due to which the GLE rich chicken patties were under the appropriate limit (Al‐dagal & Bazaraa, 1999). Our findings are consistent with Mailoa et al. (2014) who noted that GLE's antibacterial effect was significantly due to its rich tannin content.

### 3.5 Hunter color (lab)

The color preferences of meat have a significant influence on the buyer's approval. Significant changes were observed in L* value of chicken meat patties on different treatments at storage intervals. The L* value in chicken meat patties was ranged from 39.00 ± 0.32 to 55.15 ± 0.34. A higher value was observed in aerobic packed samples while a lower value was found in vacuum packed samples. However, 3% GLE‐treated samples showed high L* value as compared to all other treatments. During storage, L* value was observed to be maximum at 0 days while the lower L* color was detected at 10 days. (Table 8).

**TABLE 8:** Hunter color of chicken patties treated with gamma irradiation and guava leaf extract at different storage periods (0, 5, and 10 days).

Parameter	Irradiation dose (kGy)	Storage period (day)					
		0		5		10	
		Aerobic	Vacuum	Aerobic	Vacuum	Aerobic	Vacuum
L*	Control	47.23 ± 0.31	46.20 ± 0.28	45.21 ± 0.30	44.11 ± 0.40	39.04 ± 0.32	39.00 ± 0.32
	1% GLE	49.75 ± 0.43	48.24 ± 0.41	46.24 ± 0.25	46.07 ± 0.43	41.11 ± 0.25	41.02 ± 0.56
	2% GLE	52.01 ± 0.26	51.23 ± 0.22	49.65 ± 0.36	48.15 ± 0.30	43.20 ± 0.32	43.14 ± 0.50
	3% GLE	55.15 ± 0.34	54.06 ± 0.31	51.07 ± 0.41	50.23 ± 0.46	47.45 ± 0.42	47.12 ± 0.46
	2 kGy + 1% GLE	51.77 ± 0.21	49.65 ± 0.25	53.25 ± 0.52	52.01 ± 0.27	54.01 ± 0.28	53.45 ± 0.32
	2 kGy + 2% GLE	50.17 ± 0.12	48.23 ± 0.30	49.02 ± 0.34	47.45 ± 0.31	47.08 ± 0.30	46.02 ± 0.50
	2 kGy + 3% GLE	48.23 ± 0.32	47.21 ± 0.42	47.12 ± 0.36	46.23 ± 0.29	47.01 ± 0.30	46.01 ± 0.32
	Mean	50.61 ± 0.28a	49.26 ± 0.31b	48.79 ± 0.36a	47.75 ± 0.35b	45.55 ± 0.34	45.11 ± 0.42
a*	Control	0.20 ± 0.02	0.46 ± 0.32	1.01 ± 0.02	1.25 ± 0.10	1.38 ± 0.12	2.05 ± 0.18
	1% GLE	0.30 ± 0.03	0.38 ± 0.29	1.41 ± 0.20	2.01 ± 0.16	2.74 ± 0.15	3.15 ± 0.24
	2% GLE	0.23 ± 0.01	0.31 ± 0.25	2.46 ± 0.21	2.65 ± 0.18	2.80 ± 0.21	2.94 ± 0.21
	3% GLE	0.19 ± 0.01	0.25 ± 0.20	1.42 ± 0.01	2.20 ± 0.21	2.24 ± 0.27	2.81 ± 0.19
	2 kGy + 1% GLE	1.20 ± 0.12	1.24 ± 0.09	1.15 ± 0.06	1.79 ± 0.20	0.74 ± 0.02	2.02 ± 0.14
	2 kGy + 2% GLE	2.42 ± 0.23	2.46 ± 0.13	2.25 ± 0.12	2.78 ± 0.31	2.10 ± 0.09	3.01 ± 0.21
	2 kGy + 3% GLE	3.01 ± .26	3.42 ± 0.28	2.49 ± 0.19	3.69 ± 0.32	2.41 ± 0.13	3.88 ± 0.29
	Mean	1.07 ± 0.09b	1.22 ± 0.22a	1.74 ± 0.11b	2.33 ± 0.21a	2.06 ± 0.14b	2.84 ± 0.21a
b*	Control	1.92 ± 0.01	1.56 ± 0.02	1.98 ± 0.04	1.48 ± 0.12	2.65 ± 0.25	1.46 ± 0.09
	1% GLE	1.82 ± 0.02	1.45 ± 0.02	2.74 ± 0.13	1.26 ± 0.09	3.32 ± 0.31	1.18 ± 0.06
	2% GLE	2.05 ± 0.11	2.01 ± 0.13	2.62 ± 0.12	1.58 ± 0.08	2.97 ± 0.23	1.11 ± 0.02
	3% GLE	2.48 ± 0.18	2.39 ± 0.18	2.96 ± 0.12	2.24 ± 0.14	3.25 ± 0.32	1.98 ± 0.12
	2 kGy + 1% GLE	3.46 ± 0.31	3.24 ± 0.32	3.69 ± 0.31	2.56 ± 0.12	4.21 ± 0.41	2.33 ± 0.18
	2 kGy + 2% GLE	3.64 ± 0.34	3.54 ± 0.35	3.77 ± 0.33	3.29 ± 0.31	4.25 ± 0.40	2.96 ± 0.27
	2 kGy + 3% GLE	4.01 ± 0.40	3.88 ± 0.31	4.32 ± 0.41	3.69 ± 0.32	4.56 ± 0.42	3.27 ± 0.25
	Mean	2.77 ± 0.19a	2.58 ± 0.19b	3.15 ± 0.21a	2.3 ± 0.17b	3.60 ± 0.33a	2.04 ± 0.14b

*Note*: The values are mean ± SD of three independent determinations. Means carrying different letters in a column differed significantly (*p* < .05).

Abbreviation: GLE, guava leaf extract.

Our results showed that the a* value was reduced with increasing the % of GLE while increment was found when treated with irradiation dose as shown in Table 8. However, the a* value was measured to be high with the passage of storage time. Furthermore, the packaging materials were affected by a* value of chicken meat patties. Results showed that the vacuum‐packed samples contained a high a* amount as compared to aerobic packed samples.

The b* value in chicken meat patties ranged from 1.45 ± 0.02 to 4.56 ± 0.42 as shown in Table 8. The higher b* value was found in 2 kGy + 3% GLE while the lower value was observed in 1% GLE. During storage, with the passage of time the b* value was recorded to be high. However, at 10 days, high b* value was found while a lower value was observed at 0 days of storage. Our results showed that packaging materials affect the b* value of chicken meat patties. Moreover, maximum b* value was found in aerobic packaged samples, whereas minimum value was recorded in vacuum‐packaged samples.

The results described that the L* value significantly decreased in vacuum‐packaged chicken meat patties as compared to aerobic samples. Our results showed similarity with (Arshad, Amjad, et al., 2019) who showed that with the incorporation of TP in both aerobic and vacuum‐packed samples, the L* values in handled and undiagnosed chicken meat patties was increased. The findings showed that the processed chicken meat patties had a higher L* value in aerobic packages. Another research reported that L* values of fresh meat showed significantly (*p* < .05) higher value in Cobb400 than all indigenous breeds except Vanaraja (Pathak & Singh, 2017). The less values for L* in the controls were may be due to the melanosis exhibition (Zarehgashti et al., 2018). Arshad, Amjad, et al. (2019) reported that the redness of chicken meat increases when treated with turmeric powder which is in agreement with our study. Our findings were consistent with the results Nam and Ahn (2002) who recorded increased redness in the raw breast meat of chicken and turkey treated with irradiation. Our findings are consistent with the report of Kim et al. (2013) who reported that the yellowness (b*) in beef patties supplemented with soy sauce increased with the increase in storage interval. In addition, results showed that the yellowness (b*) of beef increased with the addition of the antioxidant source that is consistent with the results of the current study.

### 3.6 Heme pigments

Heme pigment assessment is used to evaluate meat quality parameters (Chaijan et al., 2007). Statistical findings concerning the Mb content of samples of chicken meat have a major impact on processing and storage periods.

Mb results of chicken meat patties were found to be changed significantly during storage, different treatments, and packaging. A higher value was observed on 2 kGy + 1% GLE while the minimum Mb was found on 3% GLE. However, aerobic packaged patties had more Mb value as compared to vacuum‐packaged patties. Furthermore, on both packaging, the maximum Mb amount was found at 0 days while the minimum value of Mb was observed at 10 days of storage.

The oxymyoglobin value in chicken meat patties ranged from 11.87 ± 0.45 to 24.76 ± 1.34 as shown in Table 9. The higher oxymyoglobin value was found on 2 kGy + 1% GLE while the lower value was observed on 2 kGy + 3% GLE. During storage, with the passage of time, the oxymyoglobin value was recorded to be high. However, at 10 days, high oxymyoglobin value was found while a lower value was observed at 0 days of storage. Our results showed that packaging materials affect the oxymyoglobin value of chicken meat patties. Moreover, maximum oxymyoglobin value was found in aerobic packaged samples, whereas a minimum value was recorded in vacuum packaged samples.

**TABLE 9:** Heme pigments of chicken patties treated with gamma irradiation and guava leaf extract at different storage periods (0, 5, and 10 days).

Parameter	Irradiation dose (kGy)	Storage period (day)					
		0		5		10	
		Aerobic	Vacuum	Aerobic	Vacuum	Aerobic	Vacuum
Myoglobin (%)	Control	32.45 ± 2.42	31.62 ± 2.45	24.68 ± 2.30	23.45 ± 1.40	19.21 ± 1.32	19.12 ± 0.32
	1% GLE	31.78 ± 2.34	29.15 ± 2.42	22.64 ± 1.25	20.28 ± 0.43	18.95 ± 1.25	18.05 ± 0.56
	2% GLE	29.62 ± 2.15	30.78 ± 2.50	20.12 ± 1.32	22.43 ± 1.30	18.27 ± 0.32	18.95 ± 0.50
	3% GLE	25.23 ± 1.40	28.67 ± 1.42	17.69 ± 0.41	18.23 ± 0.25	15.21 ± 0.42	17.64 ± 1.46
	2 kGy + 1% GLE	35.25 ± 2.46	33.24 ± 2.39	33.67 ± 2.58	31.68 ± 2.27	29.54 ± 1.28	28.10 ± 2.32
	2 kGy + 2% GLE	31.87 ± 2.42	32.67 ± 2.30	28.45 ± 1.34	30.29 ± 2.31	25.62 ± 1.30	26.19 ± 1.50
	2 kGy + 3% GLE	28.21 ± 1.31	29.67 ± 2.25	26.12 ± 1.36	29.23 ± 1.31	25.25 ± 2.30	26.15 ± 1.32
	Mean	30.63 ± 2.07	30.82 ± 2.25	27.62 ± 1.51a	25.08 ± 1.32b	21.72 ± 1.77a	22.02 ± 1.14a
Oxymyoglobin (%)	Control	13.23 ± 0.87	12.25 ± 0.23	16.45 ± 2.54	15.89 ± 1.54	17.67 ± 1.34	15.67 ± 1.56
	1% GLE	14.65 ± 1.23	13.78 ± 1.34	17.23 ± 2.34	15.21 ± 1.65	21.23 ± 2.32	20.34 ± 0.25
	2% GLE	15.89 ± 1.03	13.25 ± 1.21	19.65 ± 2.67	17.34 ± 2.21	22.65 ± 2.35	20.12 ± 0.23
	3% GLE	17.23 ± 2.23	15.67 ± 1.78	19.99 ± 1.95	17.99 ± 2.32	23.55 ± 2.54	21.64 ± 0.45
	2 kGy + 1% GLE	16.56 ± 1.78	14.87 ± 1.34	20.56 ± 2.43	18.23 ± 2.22	24.76 ± 1.34	21.78 ± 0.34
	2 kGy + 2% GLE	13.77 ± 0.98	11.89 ± 0.45	16.56 ± 1.23	14.45 ± 1.34	18.23 ± 0.98	15.89 ± 0.67
	2 kGy + 3% GLE	12.24 ± 0.54	11.87 ± 0.45	16.25 ± 1.67	13.11 ± 1.15	19.11 ± 1.78	15.98 ± 0.78
	Mean	14.79 ± 1.23a	13.36 ± 0.97b	18.09 ± 2.11a	16.03 ± 1.77b	21.02 ± 1.81a	18.77 ± 0.58b
Metmyoglobin (%)	Control	37.25 ± 1.54	35.23 ± 0.98	45.34 ± 2.13	42.45 ± 1.25	52.36 ± 3.65	48.65 ± 2.78
	1% GLE	40.23 ± 2.12	37.54 ± 0.78	48.12 ± 2.65	44.67 ± 2.25	52.87 ± 3.34	49.78 ± 2.65
	2% GLE	41.47 ± 2.34	37.98 ± 1.01	49.76 ± 2.39	45.23 ± 2.45	54.65 ± 2.78	51.23 ± 3.32
	3% GLE	44.56 ± 2.54	41.26 ± 1.23	51.88 ± 3.43	48.65 ± 3.02	58.12 ± 4.03	54.56 ± 3.15
	2 kGy + 1% GLE	38.67 ± 1.45	35.67 ± 1.02	44.76 ± 2.53	41.78 ± 1.32	49.34 ± 2.45	44.76 ± 1.98
	2 kGy + 2% GLE	36.54 ± 1.56	32.12 ± 0.86	43.25 ± 2.18	41.21 ± 1.25	47.54 ± 2.65	43.23 ± 2.45
	2 kGy + 3% GLE	36.21 ± 1.23	31.18 ± 1.14	41.78 ± 1.76	38.11 ± 0.67	44.43 ± 2.39	41.65 ± 1.45
	Mean	31.16 ± 1.82b	35.85 ± 1.00a	46.41 ± 2.44a	43.17 ± 1.74b	51.33 ± 3.04a	47.69 ± 2.54b

*Note*: The values are mean ± SD of three independent determinations. Means carrying different letters in a column differed significantly (*p* < .05).

Abbreviation: GLE, guava leaf extract.

Table 9 shows the result of metmyoglobin during different storage, packaging materials, and treatments. Metmyoglobin amount was observed to be enhanced with increasing the % of GLE while decrement in results was found when treated with irradiation dose. However, the metmyoglobin amount was measured to be high with the passage of storage time. Furthermore, the packaging materials affected the metmyoglobin amount of chicken meat patties. Results showed that the aerobic packed samples contained a high metmyoglobin amount as compared to vacuum‐packed samples.

The myoglobin pigment was observed to reduce according to current findings, while the oxymyoglobin and metmyoglobin pigments improved on irradiation dose and storage intervals under aerobic and vacuum packaging. This outcome coincides with the results of An et al. (2017) who indicated that during storage, heme pigments of irradiated smoked duck meat increased with the exception of myoglobin. Our findings are in accordance with the results of (Reddy et al., 2015) who claimed that the Mb oxidized into MMb with the flow of time and raise of the dose level through an intermittent MbO2 process. A previous study investigated that the presence of antioxidants in meat samples moderately prevents myoglobin deterioration during processing, thus enabling good heme pigment value in meat samples (Cunha et al., 2018).

### 3.7 Sensory evaluation of chicken patties

The sensory attributes of a product seem to be very significant. The sensory attributes play a crucial role in the demand of the market for this commodity (Sharif et al., 2017).

Average values of the color, taste, texture, odor, and overall acceptability of chicken meat patties were changed significantly. The high value of sensory parameters was found in vacuum‐packed samples, whereas the lower value of sensory parameters was observed in aerobic‐packed samples at different storage intervals (0, 5, and 10). The appearance, taste, texture, odor, and overall acceptability were in the range of 6.4 ± 0.34–7.6 ± 0.08, 5.9 ± 0.15–7.6 ± 0.45, 6.5 ± 0.20–7.8 ± 0.29, 5.4 ± 0.19–7.8 ± 0.51, and (5.2 ± 0.19–7.94 ± 0.51), respectively (Table 10).

**TABLE 10:** Sensory evaluation (appearance, texture, taste, odor, overall acceptability) of chicken meat patties treated with gamma irradiation and guava leaf extract at different storage periods (0, 5, and 10 days).

Parameter	Irradiation dose (kGy)	Storage period (day)					
		0		5		10	
		Aerobic	Vacuum	Aerobic	Vacuum	Aerobic	Vacuum
Appearance	Control	7.4 ± 0.23	7.5 ± 0.28	7.1 ± 0.24	7.0 ± 0.35	6.8 ± 0.32	6.7 ± 0.32
	1% GLE	7.3 ± 0.21	7.4 ± 0.23	7.0 ± 0.16	6.9 ± 0.26	6.6 ± 0.25	6.5 ± 0.56
	2% GLE	7.4 ± 0.23	7.5 ± 0.25	7.0 ± 0.12	7.1 ± 0.13	6.5 ± 0.32	6.4 ± 0.50
	3% GLE	7.4 ± 0.26	7.5 ± 0.07	6.9 ± 0.15	7.0 ± 0.28	6.5 ± 0.41	6.8 ± 0.23
	2 kGy + 1% GLE	7.6 ± 0.08	7.2 ± 0.18	7.4 ± 0.32	6.8 ± 0.27	7.3 ± 0.28	6.5 ± 0.29
	2 kGy + 2% GLE	7.2 ± 0.12	6.9 ± 0.09	7.1 ± 0.34	6.6 ± 0.31	7.0 ± 0.30	6.4 ± 0.34
	2 kGy + 3% GLE	7.1 ± 0.21	6.8 ± 0.12	6.9 ± 0.25	6.7 ± 0.29	6.5 ± 0.31	6.5 ± 0.32
	Mean	7.3 ± 0.13a	7.2 ± 0.17b	7.15 ± 0.22a	6.8 ± 0.27b	6.7 ± 0.31a	6.5 ± 0.38b
Texture	Control	7.4 ± 0.41	7.4 ± 0.32	7.3 ± 0.21	7.2 ± 0.25	7.1 ± 0.12	7.1 ± 0.18
	1% GLE	7.6 ± 0.32	7.8 ± 0.29	7.5 ± 0.20	7.7 ± 0.16	7.3 ± 0.34	7.5 ± 0.24
	2% GLE	7.4 ± 0.23	7.6 ± 0.25	7.3 ± 0.21	7.4 ± 0.24	7.0 ± 0.21	7.3 ± 0.21
	3% GLE	7.3 ± 0.25	7.5 ± 0.20	7.1 ± 0.34	7.3 ± 0.21	6.8 ± 0.27	7.0 ± 0.19
	2 kGy + 1% GLE	7.2 ± 0.12	7.3 ± 0.35	7.1 ± 0.28	7.1 ± 0.20	6.8 ± 0.41	6.8 ± 0.32
	2 kGy + 2% GLE	7.2 ± 0.23	7.2 ± 0.41	6.9 ± 0.12	6.8 ± 0.31	6.7 ± 0.36	6.6 ± 0.21
	2 kGy + 3% GLE	6.9 ± 0.26	7.0 ± 0.28	6.8 ± 0.23	6.8 ± 0.32	6.6 ± 0.19	6.5 ± 0.20
	Mean	7.3 ± 0.26b	7.4 ± 0.30a	7.14 ± 0.23b	7.2 ± 0.24a	6.9 ± 0.27a	6.8 ± 0.22b
Taste	Control	7.3 ± 0.42	7.6 ± 0.45	7.1 ± 0.30	7.4 ± 0.40	6.9 ± 0.32	7.3 ± 0.32
	1% GLE	6.9 ± 0.34	7.2 ± 0.42	6.7 ± 0.25	7.0 ± 0.43	6.5 ± 0.25	6.9 ± 0.56
	2% GLE	6.8 ± 0.34	7.1 ± 0.50	6.5 ± 0.32	6.8 ± 0.30	6.4 ± 0.32	6.7 ± 0.26
	3% GLE	6.7 ± 0.40	7.1 ± 0.42	6.4 ± 0.21	6.6 ± 0.25	6.3 ± 0.42	6.4 ± 0.46
	2 kGy + 1% GLE	6.6 ± 0.21	6.9 ± 0.39	6.3 ± 0.18	6.8 ± 0.27	6.2 ± 0.28	6.4 ± 0.32
	2 kGy + 2% GLE	6.6 ± 0.23	6.7 ± 0.30	6.2 ± 0.15	6.4 ± 0.23	6.1 ± 0.30	6.3 ± 0.23
	2 kGy + 3% GLE	6.4 ± 0.20	6.5 ± 0.25	6.1 ± 0.12	6.4 ± 0.31	5.9 ± 0.15	6.2 ± 0.32
	Mean	6.75 ± 0.30b	7.01 ± 0.39a	6.5 ± 0.33b	6.7 ± 0.32a	6.3 ± 0.29b	6.6 ± 0.35a
Odor	Control	7.5 ± 0.32	7.8 ± 0.51	6.8 ± 0.34	7.5 ± 0.32	6.3 ± 0.25	7.4 ± 0.41
	1% GLE	7.2 ± 0.23	7.5 ± 0.34	7.0 ± 0.41	7.4 ± 0.37	6.7 ± 0.27	7.2 ± 0.32
	2% GLE	7.1 ± 0.35	7.3 ± 0.34	6.8 ± 0.36	7.1 ± 0.24	6.5 ± 0.24	6.8 ± 0.35
	3% GLE	6.9 ± 0.25	7.2 ± 0.32	6.7 ± 0.31	6.9 ± 0.21	6.3 ± 0.23	6.6 ± 0.36
	2 kGy + 1% GLE	6.5 ± 0.23	6.9 ± 0.28	6.3 ± 0.39	6.8 ± 0.26	6.1 ± 0.21	6.5 ± 0.31
	2 kGy + 2% GLE	6.2 ± 0.15	6.7 ± 0.25	6.0 ± 0.28	6.4 ± 0.18	5.7 ± 0.18	6.2 ± 0.22
	2 kGy + 3% GLE	5.9 ± 0.19	6.3 ± 0.29	5.7 ± 0.24	6.1 ± 0.25	5.4 ± 0.19	5.8 ± 0.25
	Mean	6.7 ± 0.25b	7.1 ± 0.33a	5.5 ± 0.33b	5.81 ± 0.21a	6.14 ± 0.22b	6.6 ± 0.32a
Overall acceptability	Control	7.42 ± 0.41	7.94 ± 0.51	7.10 ± 0.35	7.62 ± 0.45	6.6 ± 0.33	7.51 ± 0.41
	1% GLE	6.95 ± 0.32	7.6 ± 0.48	6.7 ± 0.31	7.5 ± 0.41	6.1 ± 0.34	7.2 ± 0.32
	2% GLE	6.72 ± 0.31	7.1 ± 0.37	6.41 ± 0.39	6.9 ± 0.35	5.96 ± 0.19	6.6 ± 0.30
	3% GLE	6.31 ± 0.25	6.9 ± 0.34	6.04 ± 0.26	6.7 ± 0.32	5.81 ± 0.30	6.3 ± 0.28
	2 kGy + 1% GLE	6.26 ± 0.28	6.5 ± 0.32	6.0 ± 0.25	6.1 ± 0.29	5.7 ± 0.18	6.0 ± 0.21
	2 kGy + 2% GLE	5.94 ± 0.18	5.9 ± 0.28	5.8 ± 0.31	5.6 ± 0.14	5.5 ± 0.09	5.5 ± 0.28
	2 kGy + 3% GLE	5.60 ± 0.14	5.7 ± 0.23	5.4 ± 0.16	5.5 ± 0.34	5.1 ± 0.21	5.2 ± 0.19
	Mean	6.45 ± 0.27b	6.8 ± 0.36a	6.06 ± 0.04b	6.56 ± 0.33a	5.82 ± 0.23b	6.34 ± 0.28a

*Note*: The values are mean ± SD of three independent determinations. Means carrying different letters in a column differed significantly (*p* < .05).

Abbreviation: GLE, guava leaf extract.

Our findings coincide with Biswas et al. (2002) and Kumar et al. (2011) who mentioned that the taste of nuggets decreased significantly with storage time. With the passage of storage time, there was a significant decrease in all sensory parameters. The positive effect of guava leaf powder on the sensory features of processed sheep meat nuggets has been confirmed (Verma et al., 2013). Our results are in agreement with Fallah‐Araghi et al. (2012) who reported that the appearance quality of both irradiated and nonirradiated products was strong at day 1, but the nonirradiated grilled chicken showed less sensory results as compared to irradiated samples after 15 day of storage. Our result is similar to Khalid et al. (2021) who conducted research on ostrich and chicken that were treated with different doses of irradiation and kale leaf powder.

## 4 CONCLUSION

This research concluded that different treatments (guava leaf extract and gamma irradiation) preserve the chicken meat patties with minimum changes in physicochemical and sensory parameters. A decreasing trend of the total phenolic contents and antioxidant profile was evaluated during storage, but it was lower in vacuum packaging compared to aerobic packaging. The both types of packaging (vacuum and aerobic) aid in improving the shelf life of chicken patties. The 2 kGy dose reduce the total aerobic bacteria and eliminate coliform bacteria from chicken samples during time intervals (0, 5, and 10 days). The minimum changes were observed in sensory parameters of chicken patties on different treatment during storage intervals and conditions (aerobic and vacuum).

## FUNDING INFORMATION

There are no funders to report for this submission.

## CONFLICT OF INTEREST STATEMENT

All the authors have no conflict of interest in publishing this manuscript.

## Data Availability

The data will be available on request.
